# Performance Evaluation of Localization Accuracy for a Log-Normal Shadow Fading Wireless Sensor Network under Physical Barrier Attacks

**DOI:** 10.3390/s151229817

**Published:** 2015-12-04

**Authors:** Ahmed Abdulqader Hussein, Tharek A. Rahman, Chee Yen Leow

**Affiliations:** 1 Wireless Communication Centre (WCC), Faculty of Electrical Engineering, Universiti Teknologi Malaysia, UTM Skudai, Johor 81310, Malaysia; tharek@fke.utm.my (T.A.R.); bruceleow@fke.utm.my (C.Y.L.); 2 University of Technology, Baghdad 10066, Iraq

**Keywords:** average localization error, range-based received signal strength indicator (RSSI), log-normal shadow fading, physical barrier attacks, J0101

## Abstract

Localization is an apparent aspect of a wireless sensor network, which is the focus of much interesting research. One of the severe conditions that needs to be taken into consideration is localizing a mobile target through a dispersed sensor network in the presence of physical barrier attacks. These attacks confuse the localization process and cause location estimation errors. Range-based methods, like the received signal strength indication (RSSI), face the major influence of this kind of attack. This paper proposes a solution based on a combination of multi-frequency multi-power localization (C-MFMPL) and step function multi-frequency multi-power localization (SF-MFMPL), including the fingerprint matching technique and lateration, to provide a robust and accurate localization technique. In addition, this paper proposes a grid coloring algorithm to detect the signal hole map in the network, which refers to the attack-prone regions, in order to carry out corrective actions. The simulation results show the enhancement and robustness of RSS localization performance in the face of log normal shadow fading effects, besides the presence of physical barrier attacks, through detecting, filtering and eliminating the effect of these attacks.

## 1. Introduction

Wireless sensor networks serve as the link between the digital and the physical worlds. Forthcoming wireless network sensors are expected to be deployed in almost every sphere of the human activity system and used in a manner not yet envisaged. Thus, the need to mass produce low-cost integrated sensor nodes that will enhance the use of technology for bridging a myriad of domains cannot be overemphasized. Some of the few areas where future sensor networks can be deployed include earthquake monitoring, environmental monitoring, factory automation, home and office controls, inventory monitoring, medicine, *etc*. The increasing prevalence of wireless networks has not only increased the possibilities of information integration into applications, but has also influenced our interactions with others, in study and work. A typical example of such an information source is location information, which is of great importance in many applications. The term localization is the ability to determine the physical position of a static or mobile sensor node or wireless device. The use of a wireless device for tracking locations is a growing trend that holds great prospects for a wide range of applications, particularly for those employed in physical security, asset tracking, geographic routing and workflow management [1].

The increase in the use of these wireless devices by both people and objects has brought to light the importance of precision in node positioning in sensor and wireless networks. This is because of the important input role played by sensor location in various high-level networking tasks and applications. For example, nowadays, received signal strength (RSS) measurement hardware is included in all modern radio chip sets, so as to determine the transmitted packets. This comes at a very high cost and deployment benefit when the existing communication network’s RSS infrastructure is re-used for localization. The typical pattern for most RSS-based localization strategies is a transmitter or receiver that takes measurements of the RSS of a number of landmarks at known positions, also known as the device. The device is then positioned using a fingerprint method or the resulting RSS values [2].

Despite the wide variety of algorithmic strategies, such as machine learning approaches and minimization of least squares methods, proposed by different studies, the issue of multipath effects still remains a key challenge in the domain of wireless localization based on RSS. Some of the challenges range from signal blocking or shadowing, waves bouncing off an object or reflection, waves spreading as a result of obstacles or diffraction and waves bending as they pass through different mediums or refraction. All of these impact negatively on the RSS, thereby making it a tedious task to accurately determine their effects in complex indoor environments. The typical localization error averages between 10 ft to 30 ft or more for maximum errors. Furthermore, there is a tendency for the frequency and power level to negatively influence the localization performance as a result of the wireless signal propagation. The distance at which the signal can travel is dependent on the power level of the signal propagation, while the frequency of the signal transmission determines how the environment can influence the mode of the propagated signal [3].

Sensor nodes, unlike traditional networks, usually operate in attack-prone areas, thus increasing physical attack risks, which may lead to the modification of their underlying code or cryptographic material exposure. The complexity of this problem is evidenced by the inability to produce tamper-resistant sensor nodes because of the marginal cost effect of the hardware. This makes sensor nodes more vulnerable to physical attack under such environments compared to a typical personal computer located in a secure place and subjected to individual attacks from its network.

As localization is becoming popular, numerous attacks on the localization process are also on the rise. These attacks confuse the localization process and cause location estimation errors. Therefore, the ability to have efficient wireless sensor network localization accuracy in the presence of physical signal strength attacks is a challenging and extreme undertaking.

Several recent researchers were unable to accomplish a highly secured localization performance without utilizing the cryptographic technique, which requires a complex hardware design. While the main important task of the new proposed solution based on the multi-frequency multi-power localization (MFMPL) algorithm is to detect, eliminate and overcome the influence of these physical attacks (barrier attacks) under the shadow fading propagation system model without using cryptographic techniques, the new proposed solution is expected to achieve an accurate and effective performance.

## 2. Background

There are two basic ways of calculating the distance between the transmitter and the receiver in order to obtain the RSS information. One way is to convert the signal strength (SS) to a distance measurement based on the signal propagation model and then to use geometry to calculate the target nodes’ locations based on prior knowledge of the locations of the beacon nodes. This method is referred to as triangulation localization. The other way converts the RSS values based on the signal propagation behavior and the building geometry information into distance values; a method referred to as fingerprinting localization [4].

There are two basic categories of RSS-based localization methods, namely fingerprint-based methods and model-based methods. For fingerprint-based methods, the device-carrying individual needs to build a radio map at every possible location before the localization of the real-time operation, a method known as RSS profiling. The time of map building, when the links between all anchor nodes and the radio device in a network are recorded, is referred to as the offline training period. The new RSS obtained from the measurements from all of the links is then compared against the radio map, so as to select the RSS location with the closest matching as the localization result, a process referred to as the online localization period. The major limitation of the fingerprint-based method is that an extensive effort is expended in the radio map building during the training period [4].

Model-based algorithms [5] utilize a channel model with a statistical standard to produce a purposeful relationship among the RSS and distance; exploiting this purposeful relationship, the position of the sensor node is calculable from the RSS measurement from each anchor through the in-range anchor distance estimation and, after that, utilizing the lateration techniques in order to determine the sensor node position coordinates. Some relevant studies applied statistical models to produce a complete radio map for the performance of the localization, during which the position of the sensor node is evaluated straightforwardly from the RSS measurement.

In most cases of the statistical models, the relationship between the outdoor localized environment and the RSS is extremely complicated to handle, and the shadow fading has been assumed to be mutually independent, despite that environmental obstacles cause similar shadowing impacts on numerous links that pass through these obstacles. On the other side, RSS fingerprint strategies do not quite work out for the assumptions of any previous relationship between the location and RSS; however, the training stage extends important time and effort. To some degree, the training phase can be reduced by means of spatial smoothing. Yet, this is possible only for the distances of the correlated RSS. Other researchers have additionally recommended supplementing some of the estimation utilizing anticipated RSS by utilizing the channel models [6].

In summary, model-based approaches necessitate minimum training effort; however, these techniques depend vigorously on the previous information of the connection between location and RSS. On the other hand, fingerprinting approaches are not awareof any previous information about the connection between the location and RSS, yet necessitate significant time and training effort.

### 2.1. Analysis of the Range-Based Received Signal Strength Indicator Technique

The basic concepts of the RSSI ranging depict the relationship among the transmitted and received power for the wireless signals with respect to the distance of the sensor nodes. The mathematical Equation (1) indicates this relationship.

(1) $$P_{r}=P_{t}*{\left(\frac{1}{d}\right)}^{\alpha }$$

where: Pr: the wireless signal received power. Pt: the wireless signal transmitted power. d: the distance between the sending and receiving nodes. *α*: the transmission parameter value depends on the propagation environment.

By taking 10-times the logarithm of the two sides of Equation (1), then Equation (1) is changed to Equation (2).

(2) $$10\log P_{r}=10\log P_{t}-10\alpha \log d$$

Thus, 10 log p is the power expression converted to dBm. Equation (2) can be expressed according to Equation (3).

(3) $$P_{r}\left(dB\right)=A-10\alpha \log d$$

From Equation (3), the values of parameter *A* and *α* describe the relationship between the received signal strength and the distance of the signal transmission [7,8].

The RSSI propagation models currently used in wireless sensor networks include log-normal shadow models and free-space models. Log-normal shadow models are common propagation models appropriate for both indoor and outdoor environments and best suited for wireless sensor network applications, due to their adaptability to different environmental configurations. Free-space models, on the other hand, have the following advantages: (1) they have a much wider transmission distance than their carrier wavelength *α* and antenna size; (2) they do not have the problem of obstacles between their receivers and transmitters.

Assume the wireless transmission signal power is the sensor node received signal power situated at a distance of d; this can be dictated according to Equations (4) and (5).

(4) $$P_{r}\left(d\right)=\frac{P_{t}G_{t}G_{r}\lambda^{2}}{{\left(4\pi \right)}^{2}d^{2}L}$$

(5) $$PL\left(dB\right)=10\log\frac{P_{t}}{P_{r}}=-10\log\left[\frac{\lambda^{2}}{{\left(4\pi \right)}^{2}d^{2}}\right]$$

In Equation (4), $G_{t}$ and $G_{r}$ are the antenna gain, and L is the system loss parameter, which has nothing to do with the transmission. $G_{r}$ = 1, $G_{t}$ = 1 and L = 1 are usually taken. Equation (5) is the signal attenuation formula using a logarithmic expression. Received power and the distance are twice the power attenuation in Equation (5). Log-normal shadow fading is the most general propagation model. It is suitable for indoor and outdoor environments. The model gives various parameters, which can be designed for different environments, as described in Equation (6).

(6) $$PL\left(d\right)\left(dB\right)=\bar{PL}\left(d\right)+X_{\sigma }=\bar{PL}\left(d_{o}\right)+10\log\alpha \left(\frac{d}{d_{o}}\right)+X_{\sigma }$$

The parameter $d_{o}$ in Equation (6) is the near-Earth reference distance, which depends on the experiential value; the parameter *α* is a path loss index, which depends on a specific propagation environment, and its value will become larger when there are obstacles; the parameter $X_{\sigma }$ is a zero-mean Gaussian random variable. The parameters $d_{o}$, *α* and *σ* describe the path loss model, which has a specific receiving and sending distance. The model can be used for general wireless systems design and analysis [9,10]. Synthesizing the above two kinds of propagation models, the log-normal shadow model is most suitable for wireless sensor network applications, because of its universal nature and the ability to be configured according to the environments.

### 2.2. Frequency and Power Subjected Path Loss Propagation Model

The propagation model that describes the path loss can be shown as Equation (7) [10]:

(7) $$P\left(d\right)=P\left(d_{o}\right)-10\alpha \log\frac{d}{d_{o}}+X_{\sigma }$$

where P(d) refers to the received power of the wireless sensor at an exact location d to the landmark, $P(d_{o})$ is the power loss in a free space and is usually considered as one meter, *α* refers to the path loss exponent, while $X_{\sigma }$ refers to the effect of the shadowing parameter with a variance of $X_{\sigma }$, as can be found in Equation (8).

(8) $$X_{\sigma }∼N\left(0,\sigma^{2}\right)$$

The transmission power $P(d_{o})$ more often relies on the frequency utilized transmitting packets. Along these lines, $P(d_{o})$ can be considered as a transmission frequency-dependent parameter [11]. Furthermore, the path loss exponent portrays the rate of RSS changing with the distance, which has a tendency to proliferate when the device works on a higher transmission frequency. This demonstrates that the path loss exponent is relative to the transmission frequency, as shown in Equation (9) [3].

(9) $$\alpha \propto f_{\left({\mathrm{MH}}_{z}\right)}$$

At last, the shadowing $X_{\sigma }$ is the impact that causes the received signal power changes because of the obstructions in the propagation paths. It has been indicated experimentally that the shadowing is subject to the transmitted frequency and power level [12].

In most cases of the RSSI-related ranging schemes, the signal parameters of the propagation model are usually computed by the online or offline RSSI estimations between the reference nodes (beacons). Obviously, the online RSSI measurements expend computation and communication resources. Nonetheless, in practical environments, the signal propagation model is quite difficult to determine [13]. If the standard deviation and the path loss exponent are determined precisely in the network environment, at that point, the RSSI ranging is quite perfect [14].

Moreover, the RSS readings (fingerprints) are created at different locations according to the random sensor node deployment; ((xj, yj,) sj) with (xj,yj) refers to the location j where the RSS reading has been gathered as in Equation (10).

(10) $$S_{j}=\left(S_{1j}^{L_{1}},.....,S_{1j}^{L_{q}},S_{ij}^{L_{1}},.....,S_{ij}^{L_{q}},.....S_{nj}^{L_{1}},.....,S_{nj}^{L_{q}}\right)$$

This example indicates the RSS reading for n landmark and lq dimension. At each landmark, the frequency and power level combination has been defined as a dimension. We take note that certain localization schemes need preparation information (training data) to create signal maps at the offline phase, which is utilized later for the online localization process. We utilize the fingerprints gathered from the random sensor node deployment to frame the training data. In view of the propagation model of the path loss, the RSS measurements’ probability density function gathered from (N = 1,2, …N) landmarks autonomously can be composed as shown in Equation (11).

(11) $$f_{\left(x,y\right)}\left(P\right)=\prod_{i=1}^{N}\frac{10}{\log10\sqrt{2\pi }\sigma_{i}P_{i}}\exp\left[-\frac{P_{i}}{8}{\left(\log\frac{d_{i}^{2}}{\hat{d_{i}^{2}}}\right)}^{2}\right]$$

where Pi = [ p1…pN] is the received power vectors for N landmark. Additionally:

(12) $$P_{i}={\left(\frac{10\alpha_{i}}{\sigma_{i}\log10}\right)}^{2}$$

(13) $$\hat{d_{i}}=d_{o}{\left(\frac{P(d_{o})}{P_{i}}\right)}^{\frac{1}{\alpha_{i}}}$$

(x,y) is the estimated position of the unknown wireless sensor node that can be determined from the RSS measurements. Therefore, the parameter Pi consists of the path loss exponent and the variety of the shadowing, which is subject to and dependent on the frequency and power level transmission at each landmark.

## 3. Attacks Affecting Localization

Although traditional methods of cryptography, e.g., authentication, can be used to protect against conventional attacks from adversaries, like false message injections, non-cryptographic attacks are, however, completely orthogonal and have the ability to corrupt even the measurement processes. Unfortunately, traditional security services are incapable of militating against non-cryptographic attacks, thus the need to investigate the impact of these attacks on localization algorithms with the aim of proposing methods that can be effectively used to detect and eliminate such threats from the network. Despite recent advances in this domain of securing localization [15,16,17,18], there is yet to be any study in the area of robustness of RSS-based localization algorithm generation against physical attacks. This evaluative study is therefore significant in its contribution to wireless sensor network designs, particularly in the area of protocol decisions drivers, which will enable engineers to make better informed decisions about whether there is a need for a more complicated security localization algorithm.

The first step in addressing these security concerns is to look at this from the adversary’s perspective, so as to better preempt the attacks. Before a localization system’s signal strength can be attacked, the RSS readings must first be amplified or attenuated. This is achievable by attacking the transmitting device, such as using foil to cover the 802.11 card or by attacking the landmarks. Most attenuation attacks are carried out by simply placing a material between the sensors and the landmarks [19]. An adversary can also attack by amplifying or attenuating the signal strength of the RSS readings at the transmitter or receiver. A powerful attenuation loss can be achieved through the use of advanced materials, like RF-absorptive carbon fabric. The work in [20,21] presents a detailed study of propagation loss through common materials. Finally, based on the findings of this study, the removal of a barrier, such as a door, can be used to increase amplification through antenna-based methods of the corresponding material.

## 4. Related Work

A localization scheme for wireless sensor networks has been presented in [22]. This approach requires sensors to identify their location based on beacon information transmitted by locators. Each transmitted beacon contains the locator’s coordinates and the angles of the antenna boundary lines with respect to a common global axis. The communication between locators of sensors is encrypted and secure. This approach needs specialized antennae and will increase the cost of localization.

A related range-independent localization scheme, HiRLoc, has been proposed [23]. Beacon transmission is secured by using computationally-efficient cryptographic primitives in tandem with the physical medium constraints to provide localization. HiRLoc requires a directional antenna for localization, increasing the cost for localization.

The secure verification of device position scheme [24], which is based on verifiable multilateration and the measurement of radio propagation time, enhances conventional multilateration with distance estimation by verifying node positions using a set of base stations. This method requires complex time synchronization logic and extra hardware for its implementation.

The detection of malicious nodes has also received some attention, with the TSSLproposed as a solution [25]. Malicious nodes are detected in a step-wise fashion, beginning with anchor nodes collaborating by checking their coordinates, identities and time of sending information. In this proposed solution, the WSN is partitioned into sub-areas of different trust grades by using a mesh generation algorithm to segregate malicious nodes; besides, if signal strength attacks are launched, the distance estimation will be erroneous, and the error is cascaded to all successive stages.

A novel ratio-based signal strength metric (RSM) [26] has been proposed as a new solution for wireless sensor network localization. This metric directly maps information about distance to a set of landmarks with the goal to achieve robust localization in spite of attacks. However this method assumes that attacks on all of the landmarks are uniform; with variation in the attacks on the landmarks, their method performs poorly.

The improvement of localization accuracy has been proposed in [27] through applying multiple frequencies and power transmissions. By using deviations of RSS readings and residuals, the algorithm forms high quality RSS fingerprints. Although this method improved the localization accuracy, it, however, did not consider the effect of the attacks.

A scheme to ensure secure localization in the presence of cheating beacon nodes has been proposed in [28]. This method is based on known error bounds. Unfortunately, the problem with this solution is that it is built on fixing the location of beacons based on the distribution of nodes.

A proposed approach concerned with a secure location verification in [29] has been reported to be well suited to a service-restricted region. This algorithm works by considering nodes whose signal strength is incompatible with the in-region as adversaries. However, this requires deployment knowledge of all sensors, and this approach cannot be scaled to bigger networks.

## 5. Proposed Solution

We propose a solution based on multi-frequency multi-power transmission through using two techniques: a step function multi-frequency multi-power localization (SF-MFMPL) and a combination of multi-frequency multi-power localization (C-MFMPL). In order to mitigate the influence of shadow fading, the averaging of all RSS over all of the transmitted frequencies will be performed. In our solution, the anchor or access point (AP) nodes will transmit signals in multi-frequencies at multiple power levels. Sensor nodes receive these signals and construct the average RSS fingerprint from them. All of the sensors are already coded with the knowledge of the expected frequencies and the power level from the AP. By observing the RSS fingerprint that is expected, the sensor will be able to find if there is a barrier attenuating or degrading the signal. The sensor will reject the RSS fingerprints that it doubts as attenuation or degradation. Leaving those corrupted RSS fingerprints, the sensor nodes will choose the remaining RSS fingerprints from the uncorrupted RSS and utilize lateration with non-linear least squares to get the location of the sensor node.

The sensor and the AP are strictly time synchronized. This can be done by running a quartz crystal clock in all of the sensors and the AP. With the help of the quartz crystal, all of them are time synchronized. Quartz crystal-based time synchronization is easy to implement, and it is inexpensive. More information about other time synchronization methods in wireless sensor networks can be found in [30,31].

In order to avoid the estimation being affected by interference and reflection, we propose a technique to filter those signals by estimating the approximate angle of arrival of the signal. In addition, we also propose a method to get the estimation of the attack area in the network. By estimating the attack area and alerting the network administrator, those barriers can be removed and the localization accuracy improved.

### 5.1. Modified Robust Localization Based on the Multi-Frequency Multi-Power Approach

Location is achieved by utilizing a multi-frequency multi-power transmission. The knowledge of the frequency and power level from the expected antenna is known *a priori* by the sensor. We vary the frequency by three levels, 400 MHz, 600 MHz and 800 MHz, as well as synchronously vary the power level by three levels, 4 dB, 6 dB and 8 dB. After averaging each received power level that has been transmitted by the three frequency levels, three dimensions of the RSS between the sensor and each landmark result. The use of three levels of frequency and power transmission will be suitable enough to accomplish an accurate performance without any extra computational cost and complexity, as well as reducing the fingerprint database memory size.

The receiver at the sensor node will try to synchronize and check the expected power level and frequency to check if there is any signal degradation or attenuation in the path of the signal. The lateration technique is applied on the position of the sensor and the distance measurement to get the location of the sensors. In addition, based on the signal irregularities at all sensors, the map of the region of attack is found. By cooperating with all sensors, the sensor is able to identify the area of the physical barrier attack.

**Figure 1:**
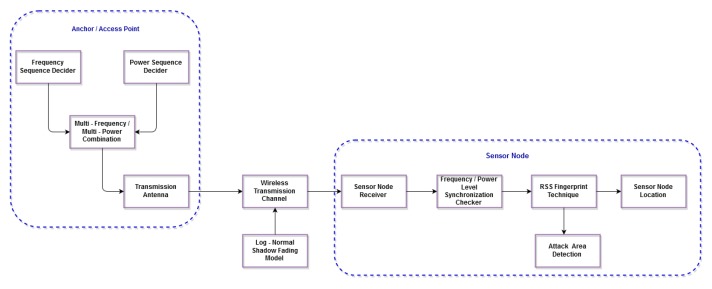
Block diagram of the proposed localization solution.

The functional block diagram of our previous work (SF-MFMPL) can be found in [32]. Figure 1 illustrates the functional block diagram of the (C-MFMPL) scheme.

The fingerprint of the proposed solution consists of two phases, offline and online. In the offline phase, the beacon signal will be checked first through various stages until the final stage, which creates the RSS fingerprint for each landmark. The sensor device is assumed to have knowledge about the AP’s position and the approximate angle of the AP’s location. Knowing the approximate angle range in which the AP is located helps to avoid any faulty measurements due to signal interference or reflection by the barriers. Since sensor nodes are placed randomly in the network, the angle of arrival of the signal from the AP is found by sensor tuning in each direction till the maximal power is received. This process has to be done for each AP at the initial deployment time. The threshold is the amount of tolerance between that estimated and the actual one. It can be measured by placing a small obstacle for signal degradation at different points in the network and measuring the error. The size of the obstacle will be the maximum obstacle size that our network can tolerate. This is done to accommodate certain infrastructure provisions made by the administrator of the network. The stage of averaging each received power level that has been transmitted by the three frequency levels is very important to overcome the effect of the log-normal shadow fading effects. Finally, the last stage in the offline phase is the cooperation of the sensor nodes to create the RSS fingerprint database of the whole network, while in the online phase, the sensor node receives the beacon signal, then averages the RSS and matches it to the fingerprint database, besides using the lateration and trilateration techniques to pin-point the sensor node position.

**Figure 2:**
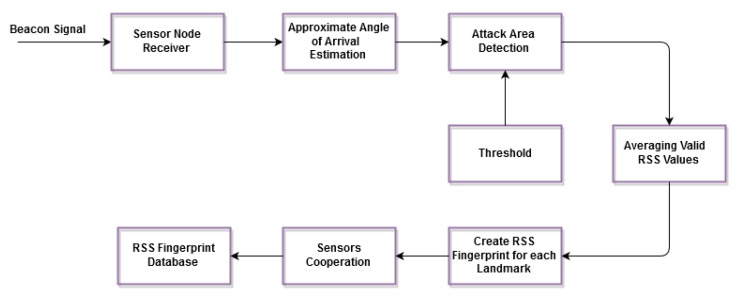
Offline phase of the proposed solution.

1. Offline Phase: 

The fingerprints are produced and stored in a database during this phase containing the average RSS readings of the anchor nodes’ positions. Actually, the location of the anchor nodes ought not to change during the online phase. Figure 2 shows the block diagram of the offline phase.

The representation of the fingerprint database for the (C-MFMPL) solution can be utilized by a set of RSS with the coordinates of the anchor nodes gathered by each sensor in the network, as shown in Equation (14).

(14) $$F=\left\{f_{1}\left(P_{1},P_{2},P_{3}\right),f_{2}\left(P_{1},P_{2},P_{3}\right),f_{3}\left(P_{1},P_{2},P_{3}\right)\right\}$$

where: f1, f2, f3 are the transmitted frequencies; P1, P2, P3 are the decided power levels.

The average RSS all over the transmitted frequencies that are stored in the fingerprint database can be defined in Equations (15) and (16), respectively.

(15) $$S_{offline}=\left\{averageRSS_{1}\left(f_{1},f_{2},f_{3}\right),averageRSS_{2}\left(f_{1},f_{2},f_{3}\right),averageRSS_{3}\left(f_{1},f_{2},f_{3}\right)\right\}$$

(16) $$S_{offline}=\left\{r_{i}^{j=1},r_{i}^{j=2},......,r_{i}^{j=m}\right\}$$

$r_{i}^{j}$ represents the average RSS readings estimated from anchor j, and m represents the number of anchors utilized in the network.

Moreover, Equation (17) shows the $R_{offline}$ set that is gathered and stored in the fingerprint database during the offline phase.

(17) $$R_{offline}=\left\{P_{offline},S_{offline}\right\}$$

The $P_{offline}$ and $S_{offline}$ sets represent the anchor nodes’ coordinates and the average RSS measurements, respectively.

While in the (SF-MFMPL) solution, each anchor (AP) sends beacons according to a step function, each decided power level will be sent with a decided frequency [32]. In order to mitigate the effect of the log-normal shadow fading, the averaging of N (RSS) values collected from each anchor (AP) has been performed in this solution according to Equation (18).

(18) $$averageRSSI_{i}=\frac{1}{N}\sum_{j=1}^{N}RSSI_{ij}$$

The value of (N = 3) will be taken for this solution to maintain the same system model conditions that are used for the (C-MFMPL) technique.

**Figure 3:**
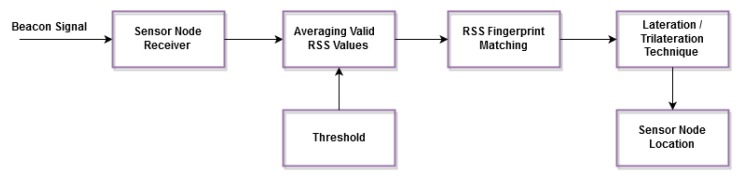
Online phase of the proposed solution.

2. Online Phase: 

During the online phase, the sensor nodes receive the transmitted beacons from the anchors with the same combinations of the frequency and power levels. Figure 3 illustrates the online phase of the proposed solution.

Therefore, another measurement set of the RSS, called $S_{online}$, is produced, which will be utilized for seeking the right fingerprints according to the fingerprint matching database, as shown in Equations (19) and (20), respectively.

(19) $$S_{online}=\left\{R_{online}\right\}$$

(20) $$R_{online}=\left\{r_{i}^{j=1},r_{i}^{j=2},......,r_{i}^{j=m}\right\}$$

$R_{online}$ denotes the average RSS readings received from each anchor node.

Eventually, the online phase depends on the RSS fingerprints generated by the offline phase to find the best matching RSS values for the estimation of the sensor node location. The RSS fingerprint machine learning database plays the most important key role in the proposed algorithm, first through filtering the physical barrier attack effects with the help of the proposed grid coloring algorithm, which depends on the angle of arrivals of the beacon signals received from each anchor (AP), and, second, participating in the process of mitigating the harsh shadow fading environment.

### 5.2. RSS Measurement

As an aspect of sensor network communication and sensing subsystems, various sensor designs have been supported by the angle of arrival (AOA) mechanism, such as tracking and other applications, which accord improved monitoring in the orientation of the target by means of beamforming [33]. Sensors might also be supported by directional antenna arrays, so that the communication transmission can be directed toward the required destination and lessening the neighbor sensor interference. With the antenna beam pattern information, a single radio receiver with a multiple antennae system might also be utilized for the efficient angle of arrival determination of the RF signal.

A multiple beam pattern of the directional antenna array can be used to determine the received signal strength RSS ratio. This solution establishes a low complexity and cost for the sensor network design. Each anchor (AP) is assigned a combination of the frequency and power level transmission. Before starting localization, each sensor node tunes in to the approximate angle in which an AP is located to capture the signal and measure the RSS. By tuning in to the approximate angle, we are able to filter out the RSS measurement errors due to reflection and interference signals from other directions. Each sensor calculates the estimated signal frequency and power according to the defined frequency and power level combination. Depending on Equations (21) and (22), the tuning is done in a way to minimize the error between the actual transmitted frequency and power level.

(21) $$Ef=abs(y_{f}-y_{af})$$

(22) $$Ep=abs(y_{p}-y_{ap})$$

If the deviation in the error within the approximate angle is greater than the threshold, the RSS value from that AP must be rejected, as it is an indication of signal attenuation or degradation by the attacker; the deviation in error can be calculated from Equations (23) and (24), respectively.

(23) $$Df=min\left(Ef\right)\;\;\;\;\;\;\;\;\;\;\;\;\;\;\theta_{min}<\theta <\theta_{max}$$

(24) $$Dp=min\left(Ep\right)\;\;\;\;\;\;\;\;\;\;\;\;\;\;\theta_{min}<\theta <\theta_{max}$$

*Df* and *Dp* must be less than threshold T for the RSS value in order to be accepted. Every sensor must measure the average RSS value all over the transmitted frequencies for the same anchor (AP). The best value of the RSS with the lower values of *Df* and *Dp* must be taken. The AP’s location (x, y) together with its measured average RSS value constitutes the RSS fingerprint for that AP. Each sensor node must calculate the average RSS fingerprint for all of the APs whose *Df* and *Dp* is less than the threshold T.

In this paper, The RSS error between the original measurements and the reconstructed measurements at a certain threshold, which presents the least amount of error for an individual network to be localized, is identified by the RSS error threshold value. Intuitively, as the discard threshold increases, the error increases (that is, the ability to reconstruct a true representation of the original signal degrades), for which the RSS error from each anchor (AP) increasing by more than 15% of the nominal RSS will be neglected, while RSS error less than 15% will be accepted. In order to select an appropriate RSS threshold, the use of an exhaustive search based on computer simulations is mandatory, due to the complexity involved in the problem at hand. Meanwhile, finding the optimum threshold value is difficult and needs extensive training for the simulation procedure. Hence, based on the relevant empirical and experimental studies related to the threshold value selection, we consider the RSS error up to 15% of the nominal RSS as the acceptable approximate threshold.

### 5.3. Localization

Localization involves two stages: ranging and lateration. Ranging estimates the distance d from the position of the sensor node to the anchor (AP). The RSS can be expressed according to Equation (25).

(25) $$RSS=P_{o}-10\alpha \log d$$

Therefore, the distance can be determined from Equation (26) as follows:

(26) $$d=10{(}^{\frac{P_{o}-RSS}{10\alpha }})$$

where: $P_{o}$ is the power received in dBm at a 1-m distance; d is the distance between the node and the AP; *α* is the path loss component.

Lateration gets the position of the sensor node from the distance measurement for different anchors (AP) and their locations. There are two popular methods to get the location estimate: non-linear least squares (NLS) and linear least squares (LLS). In NLS, from the estimated distance di and known positions Li = (xi,yi) of the landmarks, the position (x,y) of the target device can be estimated by finding $\left(\hat{x},\hat{y}\right)$ satisfying Equation (27).

(27) $$\left(\hat{x},\hat{y}\right)=\arg min_{x,y}\sum_{i=1}^{n}{\left[\sqrt{{\left(xi-x\right)}^{2}+{\left(yi-y\right)}^{2}}-\hat{d_{i}}\right]}^{2}$$

where i = 1…n for n total landmarks.

Since solving this equation is computationally complex, we can approximate this relation as shown in Equation (28) [34].

(28) $$A\hat{P}=b$$

where:

(29) $$A=\left(\begin{array}{cc} x1-\frac{1}{n}\sum_{i=1}^{n}xi & xn-\frac{1}{n}\sum_{i=1}^{n}xi\\ y1-\frac{1}{n}\sum_{i=1}^{n}yi & yn-\frac{1}{n}\sum_{i=1}^{n}yi \end{array}\right)$$

and:

(30) $$b=\frac{1}{2}\left(\begin{array}{c} \left(x_{1}^{2}-\frac{1}{n}\sum_{i=1}^{n}x_{i}^{2}\right)+\left(y_{1}^{2}-\frac{1}{n}\sum_{i=1}^{n}y_{i}^{2}\right)\\ \left(x_{n}^{2}-\frac{1}{n}\sum_{i=1}^{n}x_{i}^{2}\right)+\left(y_{1}^{2}-\frac{1}{n}\sum_{i=1}^{n}y_{i}^{2}\right)-\left({\hat{d}}_{n}^{2}-\frac{1}{n}\sum_{i=1}^{n}{\hat{d}}_{i}^{2}\right) \end{array}\right)$$

A is described by the coordinates of landmarks, and b is composed of the estimated distance to landmarks. The position estimation is solved by Equation (31).

(31) $$\hat{P}={\left(A^{T}A\right)}^{-1}A^{T}b$$

Moreover, for the localization based on a trilateration technique, the anchor nodes’ coordinates defined by the i-th beacon Bi are (xBi, yBi); the coordinates of the unknown sensor node are (x,y); RSSIi refers to the signal strength measurements between the sensor node and each anchor node; and di is the distance estimation among the sensor node and the i-th beacon node Bi. Assume three beacons (b1, b2, b3) with three distance estimations (d1, d2, d3), then the sensor node coordinates can be obtained as follows [14]:

(32) $$\begin{array}{cc} {\left(x-x_{B1}\right)}^{2}+{\left(y-y_{B1}\right)}^{2}=d_{1}^{2} & \\ {\left(x-x_{B2}\right)}^{2}+{\left(y-y_{B2}\right)}^{2}=d_{2}^{2} & \\ {\left(x-x_{B3}\right)}^{2}+{\left(y-y_{B3}\right)}^{2}=d_{3}^{2} \end{array}$$

By utilizing the earlier two equation minus the third one, respectively, we can determine the following equations:

(33) $$\begin{array}{cc} 2\left(x_{B3}-x_{B1}\right)x+2\left(y_{B3}-y_{B1}\right)y & =x_{B3}^{2}-x_{B1}^{2}+y_{B3}^{2}-y_{B1}^{2}+d_{1}^{2}-d_{3}^{2}\\ 2\left(x_{B3}-x_{B2}\right)x+2\left(y_{B3}-y_{B2}\right)y & =x_{B3}^{2}-x_{B2}^{2}+y_{B3}^{2}-y_{B2}^{2}+d_{2}^{2}-d_{3}^{2} \end{array}$$

By expressing Equation (33) in matrix form, we get:

(34) $$\left[\begin{array}{c} x\\ y \end{array}\right]={\left[\begin{array}{cc} 2(x_{B3}-x_{B1}) & 2(y_{B3}-y_{B1})\\ 2(x_{B3}-x_{B2}) & 2(y_{B3}-y_{B2}) \end{array}\right]}^{-1}\cdot \left[\begin{array}{c} x_{B3}^{2}-x_{B1}^{2}+y_{B3}^{2}-y_{B1}^{2}+d_{1}^{2}-d_{3}^{2}\\ x_{B3}^{2}-x_{B2}^{2}+y_{B3}^{2}-y_{B2}^{2}+d_{2}^{2}-d_{3}^{2} \end{array}\right]$$

Along these lines, rather than using the real distance di, it is sensible to utilize the noisy estimations $\hat{d_{i}}$, then Equation (34) can be denoted as follows:

(35) $$\begin{array}{cc} {\left(\hat{x}-x_{B1}\right)}^{2}+{\left(\hat{y}-y_{B1}\right)}^{2}={\hat{d}}_{1}^{2} & \\ {\left(\hat{x}-x_{B2}\right)}^{2}+{\left(\hat{y}-y_{B2}\right)}^{2}={\hat{d}}_{2}^{2} & \\ {\left(\hat{x}-x_{B3}\right)}^{2}+{\left(\hat{y}-y_{B3}\right)}^{2}={\hat{d}}_{3}^{2} \end{array}$$

(36) $$\left[\begin{array}{c} \hat{x}\\ \hat{y} \end{array}\right]={\left[\begin{array}{cc} 2(x_{B3}-x_{B1}) & 2(y_{B3}-y_{B1})\\ 2(x_{B3}-x_{B2}) & 2(y_{B3}-y_{B2}) \end{array}\right]}^{-1}\cdot \left[\begin{array}{c} x_{B3}^{2}-x_{B1}^{2}+y_{B3}^{2}-y_{B1}^{2}+{\hat{d}}_{1}^{2}-{\hat{d}}_{3}^{2}\\ x_{B3}^{2}-x_{B2}^{2}+y_{B3}^{2}-y_{B2}^{2}+{\hat{d}}_{2}^{2}-{\hat{d}}_{3}^{2} \end{array}\right]$$

where $\left(\hat{x},\hat{y}\right)$ is the unknown node coordinate estimation. By subtracting Equation (36) from Equation (34), we can get the localization error of the unknown sensor node as below:

(37) $$\left[\begin{array}{c} \hat{x}-x\\ \hat{y}-y \end{array}\right]={\left[\begin{array}{cc} 2(x_{B3}-x_{B1}) & 2(y_{B3}-y_{B1})\\ 2(x_{B3}-x_{B2}) & 2(y_{B3}-y_{B2}) \end{array}\right]}^{-1}\cdot \left[\begin{array}{c} \left({\hat{d}}_{1}^{2}-d_{1}^{2}\right)-\left({\hat{d}}_{3}^{2}-d_{3}^{2}\right)\\ \left({\hat{d}}_{2}^{2}-d_{2}^{2}\right)-\left({\hat{d}}_{3}^{2}-d_{3}^{2}\right) \end{array}\right]$$

In view of Equation (37), it demonstrates the localization error for utilizing the trilateration method, which is identified with both the RSSI ranging errors and the anchor node coordinates, as it is known *a priori* that the anchor nodes are fixed nodes with known coordinates, so that the localization error is resolved in terms of the RSSI ranging error only, besides the anchor nodes having to be deployed legitimately. Along these lines, the error in the localization accuracy can be determined by the equation below:

(38) $$Localization\;\;Error\;\;\;\left(E\right)=\left[\begin{array}{c} \left({\hat{d}}_{1}^{2}-d_{1}^{2}\right)-\left({\hat{d}}_{3}^{2}-d_{3}^{2}\right)\\ \left({\hat{d}}_{2}^{2}-d_{2}^{2}\right)-\left({\hat{d}}_{3}^{2}-d_{3}^{2}\right) \end{array}\right]$$

### 5.4. Detecting Attack-Prone Regions

One of the important advantages of our solution is that we can detect the region of attack and take some corrective action. In our solution, each sensor node can identify the AP that has barriers in the path of the sensor node. Each sensor node sends data to a signal fusion center, which marks the boundary of the attack region and handles it by eliminating barriers effect.

**Figure 4:**
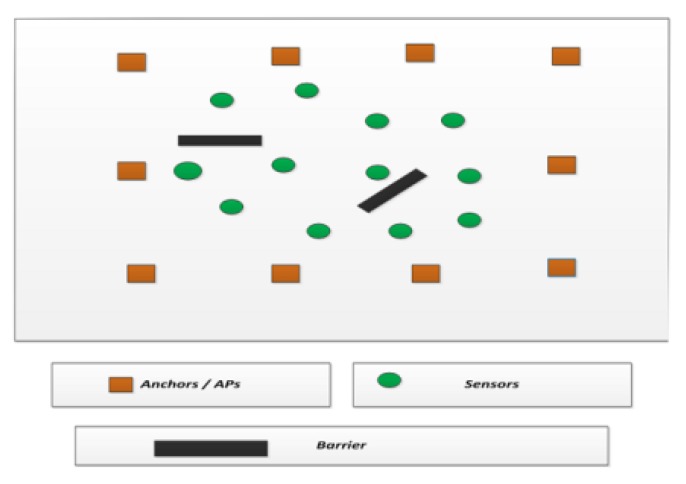
Wireless sensor network system model with APs, sensors and the barriers.

Figure 4 shows a network with APs, sensors and barriers. With the accepted AP (whose error deviation is less than the threshold) and the rejected AP (whose error deviation is above the threshold), the sensor node location estimated is provided to the signal fusion center. The signal fusion center is also aware of the direction of the propagation of the signal from the anchor (AP) to the sensor nodes.

**Figure 5:**
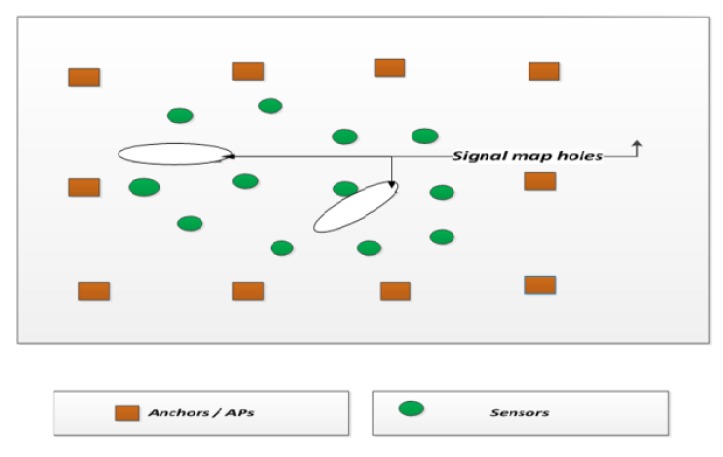
Signal map holes at the fusion center.

Based on this information, the signal fusion center constructs a signal map. The signal map is the representation of points where the signals from the APs are able to traverse the network. Signal map holes are places where the signal from the AP is not accepted at the sensor node due to a larger error deviation in the signal power, as shown in Figure 5.

Actually, sensor nodes have the flexibility to communicate with the anchor node, so as to build the radio signal map by means of the wireless channel. As a rule, a fusion center at each sensor node is jointly processing the signal in order to make accurate situational estimations. For the detection part, the fusion center necessitate making a decision concerning the presence of an attenuation of the received signal strength due to a barrier attack. Typically, a threshold value should be utilized to make a proper decision, and it is a significant parameter for the detection procedure.

Based on the whole network signal map defined by the RSS fingerprint database, which is created by the cooperating sensor nodes, actually, the valid RSS readings will be utilized for the estimation of the sensor node location, while the neglected RSS values refer to the signal holes in the fingerprint to be taken into consideration for detecting the area of attack. This mechanism introduces the basis of the fusion center at each sensor node to make the proper decision for identifying the signal holes in the network.

Through construction of the signal map, we are able to identify the signal holes in the network. Signal holes are the regions of attack in the network and must be corrected. The usual corrective action is to remove the barrier manually.

To detect the signal hole, we propose a grid coloring algorithm. The algorithm splits the entire network into small equally-sized grids. Initially, all of the grids are white. The sensor locations are marked in the grids. When a sensor accepts the AP’s RSS value (since the error deviation in the signal power is less than the threshold), the grids in the direction from the AP to the sensor node along the direction of the signal are colored gray. This process is performed for all sensors for all of the accepted AP. Once the process is complete, all of the grids still in white are the signal holes.

The algorithm code is given below:

**Algorithm 1** Signal hole detection.**Require:** GM[][]←SplitnetworkintosmallgridsN*N  **for** i=1: N **do**    **for** j=1: N **do**      GM[i][j] = White    **end for**  **end for**  **for** i=1: NoofSensor **do**    **for** j=1:NoofAP **do**      **if** error_deviation(AP)<threshold **then**        **for** X=1: N **do**          **for** Y=1: N **do**            **if** Grid(X,Y)isindirectionofAP,sensor(i) **then**              GM(X,Y)←gray            **end** **if**          **end for**        **end for**      **end if**    **end for**  **end for**

Error in the estimated attack area is calculated by Equation (40).

(39) $$E=nG_{Act}-nG_{F}$$

Error in the estimated attack area is calculated by Equation (40).

(40) $$E=nG_{Act}-nG_{F}$$

where: $nG_{Act}$ is the No. of gray squares actually in the attack area; $nG_{F}$ is the No. of gray squares estimated in the attack area.

## 6. Performance Analysis

We simulate the proposed solution by using MATLAB. The simulation area consists of a 1000 × 1000 m two-dimensional terrain. The optimal number and placement of the anchor (AP) is important. We assume a small, but reasonable beacon node population of 20 beacon nodes (approximately five beacons in each direction), which are scattered uniformly over the 1000 m × 1000 m areas. We place a maximum of 10 barriers of a length of 1 m and a width of 1 m randomly in the network. The barriers can cause signal strength degradation and have a severe effect on the RSS. This section presents the performance evaluation of the localization accuracy for both of the proposed solutions SF-MFMPL and C-MFMPL in the face of the log-normal shadow fading environment. Table 1 shows the system model specification.

**Table 1:** System model specifications.

System Model	Simulation Specifications
Network Area	1000 × 1000 m
Frequency Used by AP	400, 600, 800 MHz
Power Used by AP	4, 6, 8 dB
Barrier dimension	1 × 1 m
Sensor Node Placement	Random
AP Placement	Around Perimeter
Propagation Model	Log-Normal Shadow Fading Model

It is essential for an examination to assess all outline and advancement works with respect to the research goals. In this way, the performance evaluation is given with respect to this issue. The performance evaluation is performed according to two main parts: first, the localization accuracy, which is defined by the average localization error for different parameters of the network system model, as well as different shadow fading propagation models, and the second part is the detection efficiency of the physical signal strength attacks, which is performed according to the detected area of attacks error.

The average localization error has been achieved according to two cases:

Case I: path loss exponent *α* = 3, standard deviation of the log-normal shadow fading *σ* = 5 dB;

Case II: path loss exponent *α* = 4, standard deviation of the log-normal shadow fading *σ* = 6 dB.

**Figure 6:**
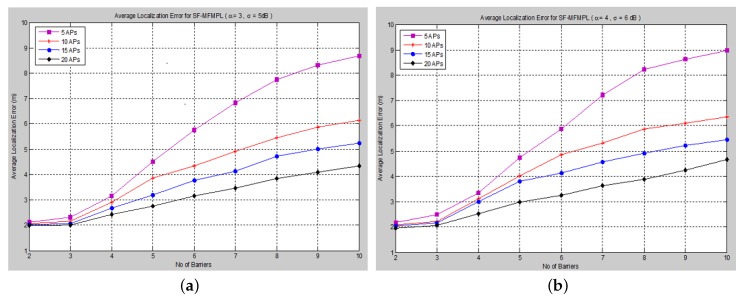
Average localization error *versus* the number of barriers for the step function multi-frequency multi-power localization (SF-MFMPL) algorithm. (**a**) Lateration technique Case I; (**b**) Lateration technique Case II.

**Figure 7:**
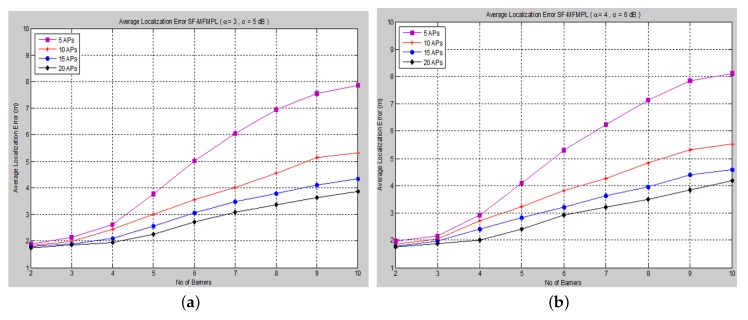
Average localization error *versus* the number of barriers for the SF-MFMPL algorithm. (**a**) Trilateration technique Case I; (**b**) Trilateration technique Case II.

The average localization error is calculated between the actual and the estimated locations for all sensor nodes. The performance is measured in terms of average localization error by varying the number of barriers in the network for different numbers of APs and for both propagation system model cases. Figure 6 and Figure 7 show the average localization error for the (SF-MFMPL) specified by the lateration and trilateration method, respectively.

It is surely understood that expanding the density of anchor nodes accordingly improves localization. However, increasing the number of anchor nodes may not be a feasible solution due to the extra hardware requirements, which may be more expensive.

The simulation results in Figure 6 and Figure 7 show an efficient localization accuracy with respect to the few and reasonable number of anchor nodes that have been applied.

**Figure 8:**
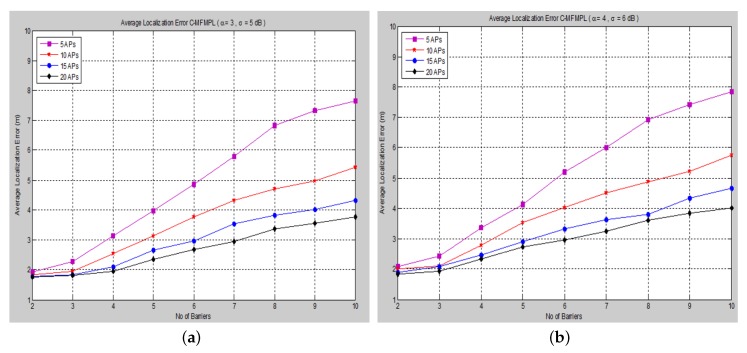
Average localization error *versus* the number of barriers for the combination of multi-frequency multi-power localization (C-MFMPL) algorithm. (**a**) Lateration technique Case I; (**b**) Lateration technique Case II.

**Figure 9:**
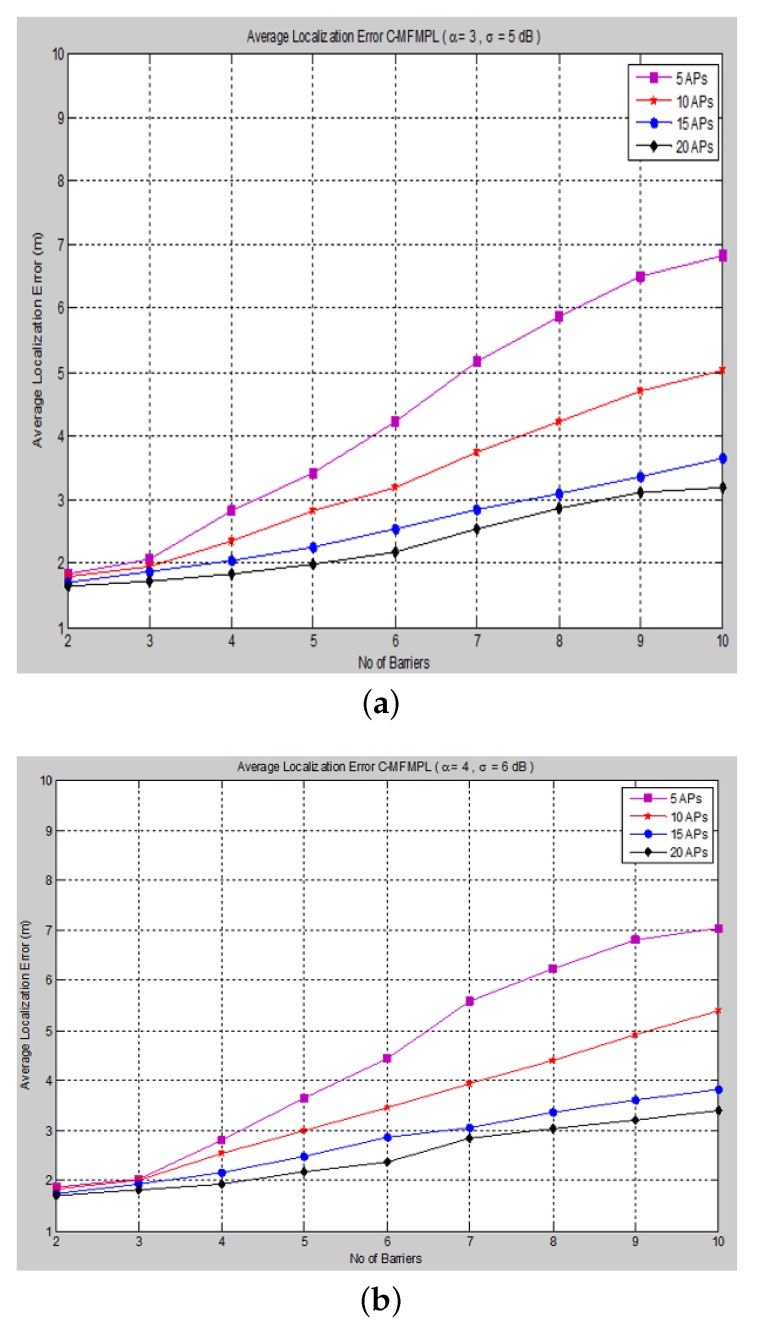
Average localization error *versus* the number of barriers for the C-MFMPL algorithm. (**a**) Trilateration technique Case I; (**b**) Trilateration technique Case II.

Moreover, Figure 8 and Figure 9 show the average localization error for the (C-MFMPL) specified by the lateration and trilateration methods, respectively.

From the simulation results, we observe that the number of anchors (APs) is a significant factor and plays a key role affecting the localization accuracy. Hence, in our approach, the increasing of the number of the anchors (APs) for the system model will lead to reducing the localization error very rapidly, because the number of variables used in non-linear regression increases, as well. On the other hand, utilizing the trilateration technique enhances the performance of the localization clearly, as compared to the performance based on lateration.

**Figure 10:**
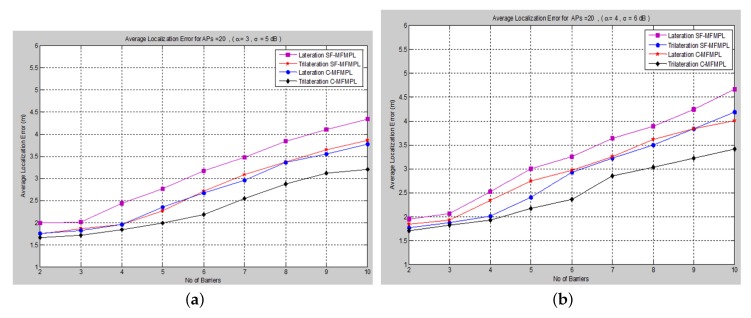
A comparison of the average localization error for both algorithms, (SF-MFMPL) and (C-MFMPL), respectively. (**a**) Case I; (**b**) Case II.

A comparison of the average localization error for both the (SF-MFMPL) and (C-MFMPL) algorithms according to 20 anchors (APs) and two propagation model cases is shown in Figure 10.

With regards to the simulation results above, we observe that for the localization accuracy identified by the average localization error for both proposed solutions, C-MFMPL is better than the proposed solution based on SF-MFMPL in both cases of the shadow fading propagation model, as well as the lateration and trilateration techniques. This is due to different strategies of using multi-frequency and multi-power for the two algorithms. Meanwhile, the propagation parameters *α* and *σ* are clearly subject to and depend on the transmission frequency and power level. In (SF-MFMPL), each power level is indicated by a specific transmitted frequency, while in (C-MFMPL), a combination of multiple frequencies and power levels has been indicated. Hence, the difference in the two algorithms’ strategies will lead to different results in the face of the impact of shadow fading. In addition, simulation results of the trilateration technique showed the best performance for both proposed solutions and under various simulation conditions. Table 2 illustrates a comparison summary of the average localization error for the two proposed solutions according to 20 anchors (APs) and two propagation model cases.

**Table 2:** Average localization error comparison of the two proposed solutions according to 20 anchors (APs) and two propagation model cases.

Propagation Model Case	Average Localization Error (m) SF-MFMPL		Average Localization Error (m) C-MFMPL	
	Lateration	Trilateration	Lateration	Trilateration
**Case I**	4.34	3.86	3.77	3.20
**Case II**	4.67	4.19	4.01	3.41

**Figure 11:**
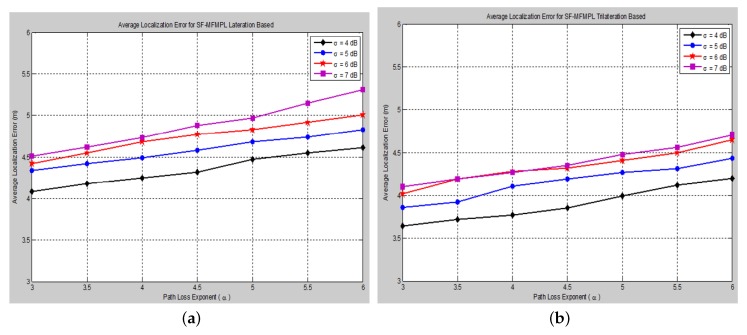
Average localization error *versus* different path loss exponent values for the SF-MFMPL algorithm (No. of barriers = 10, APs = 20). (**a**) Lateration technique based; (**b**) Trilateration technique based.

**Figure 12:**
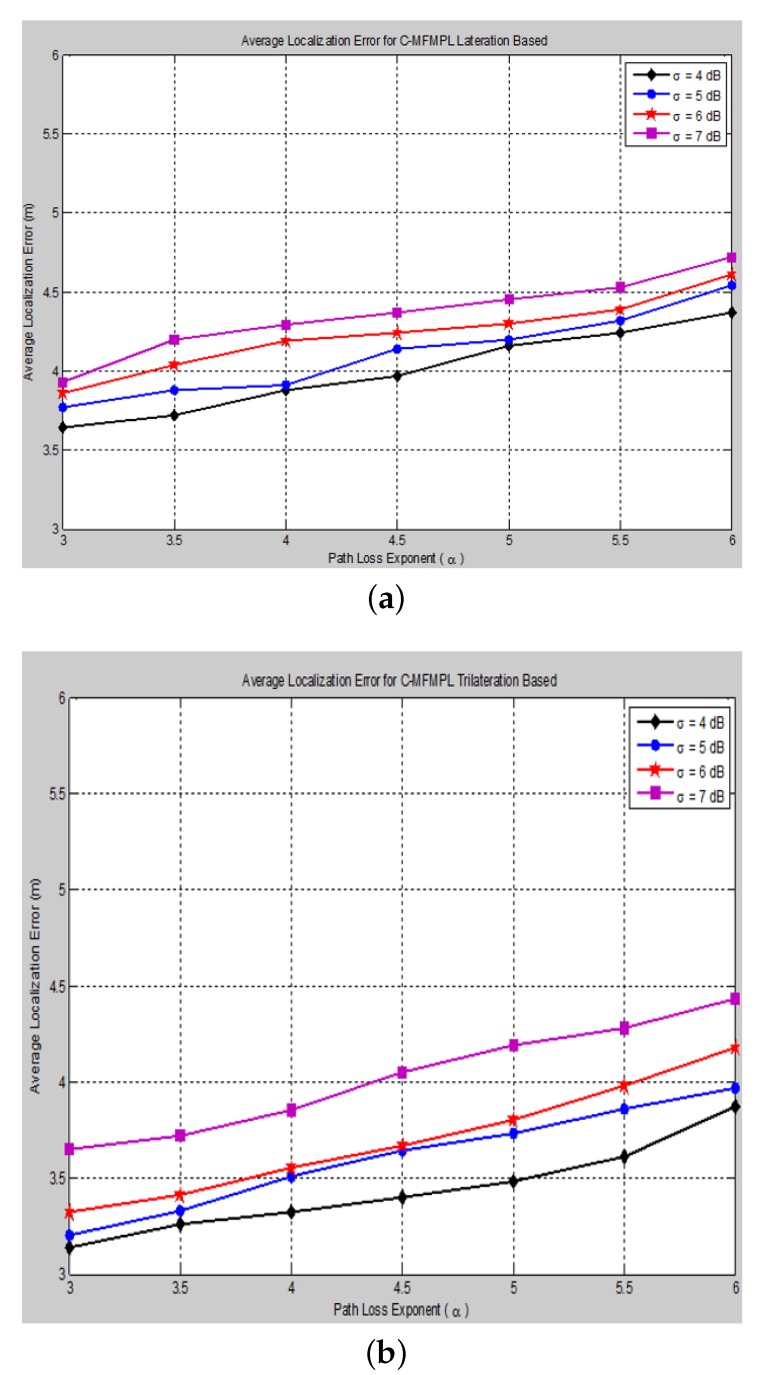
Average localization error *versus* different path loss exponent values for the C-MFMPL algorithm (No. of barriers = 10, APs 20). (**a**) Lateration technique based; (**b**) Trilateration technique based.

The effect of changing the path loss exponent (*α*) on the average localization error with respect to different values of the log-normal shadow fading standard deviation (*σ*) has been taken into consideration of the simulation analysis for both proposed algorithms, (SF-MFMPL) and (C-MFMPL), as shown in Figure 11 and Figure 12, respectively.

In practice, the shadow fading propagation model parameters are unpredictable; therefore, it will be susceptible to errors; therefore, we considered the influence of changing the propagation model parameters identified by the path loss exponent and the standard deviation on the localization accuracy for both of our proposed solutions. As illustrated in the figures above, our proposed algorithms showed an efficient and accurate performance for the estimation of the sensor nodes’ positions with an acceptable localization error. In addition, the proposed solution based on (C-MFMPL) has a better performance than the (SF-MFMPL) solution in both cases of lateration and trilateration. Table 3 and Table 4 indicate a comparison of the performance evaluation for our proposed solutions with respect to the changing of the shadow fading propagation parameters based on the lateration and trilateration techniques, respectively.

**Table 3:** Average localization error comparison of the two proposed solutions according to changing the shadow fading propagation parameters (lateration based).

Path Loss Exponent (*α*)	Average Localization Error (m)							
	SF-MFMPL Lateration Based				C-MFMPL Lateration Based			
	*σ* = 4	*σ* = 5	*σ* = 6	*σ* = 7	*σ* = 4	*σ* = 5	*σ* = 6	*σ* = 7
***α* = 3**	***4.08***	***4.34***	***4.42***	***4.51***	***3.64***	***3.77***	***3.86***	***3.93***
***α* = 4**	***4.25***	***4.49***	***4.68***	***4.73***	***3.88***	***3.91***	***4.19***	***4.29***
***α* = 5**	***4.47***	***4.68***	***4.84***	***4.97***	***4.16***	***4.20***	***4.30***	***4.44***
***α* = 6**	***4.61***	***4.83***	***5.01***	***5.31***	***4.37***	***4.54***	***4.61***	***4.72***

**Table 4:** Average localization error comparison of the two proposed solutions according to changing the shadow fading propagation parameters (trilateration based).

Path Loss Exponent (*α*)	Average Localization Error (m)							
	SF-MFMPL Trilateration Based				C-MFMPL Trilateration Based			
	*σ* = 4	*σ* = 5	*σ* = 6	*σ* = 7	*σ* = 4	*σ* = 5	*σ* = 6	*σ* = 7
***α* = 3**	***3.64***	***3.86***	***4.02***	***4.10***	***3.14***	***3.20***	***3.32***	***3.65***
***α* = 4**	***3.77***	***4.11***	***4.28***	***4.28***	***3.32***	***3.51***	***3.55***	***3.85***
***α* = 5**	***3.99***	***4.27***	***4.41***	***4.48***	***3.47***	***3.73***	***3.81***	***4.19***
***α* = 6**	***4.20***	***4.43***	***4.65***	***4.71***	***3.87***	***3.97***	***4.18***	***4.44***

**Figure 13:**
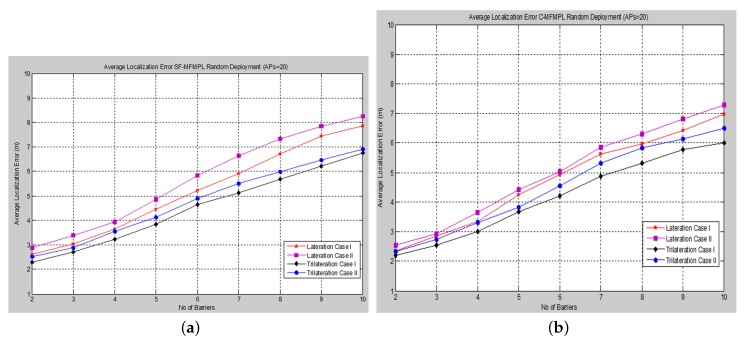
Localization error *versus* the number of barriers for a random placement configuration of APs. (**a**) SF-MFMPL; (**b**) C-MFMPL.

The other cause of localization error arises due to the anchor node’s position. We considered the effect of the anchor node’s positions on localization accuracy. Hence, the anchor node’s placement and geometry have a crucial role to play with respect to localization performance. The average localization error for the random anchor placement configuration is measured for both the (SF-MFMPL) and (C-MFMPL) algorithms, respectively, as illustrated in Figure 13.

According to the simulation results above, it is clearly observed that the localization accuracy in the case of the random deployment of the anchor nodes (APs) drops slightly with respect to the increase of the number of barriers in the network. However, the average localization error for this case is still within the acceptable error range regarding the RSSI-based localization technique.

**Figure 14:**
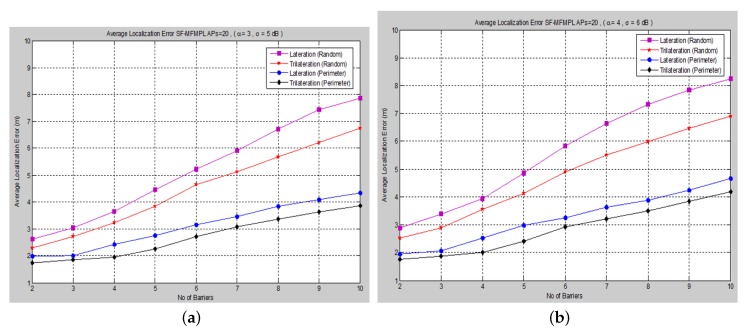
Localization error *versus* the number of barriers for SF-MFMPL with a random, around the perimeter placement configuration of APs. (**a**) Case I; (**b**) Case II.

**Figure 15:**
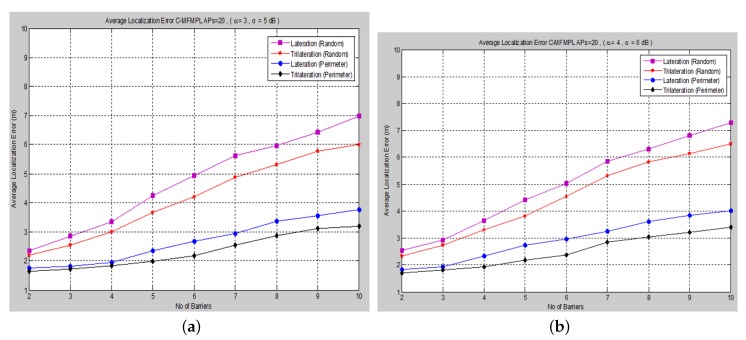
Localization error *versus* the number of barriers for C-MFMPL with a random, around the perimeter placement configuration of APs. (**a**) Case I; (**b**) Case II.

Proper positioning of anchor nodes is necessary for the effective localization accuracy of wireless sensor networks. Since the performance of the localization accuracy depends on the placement of the anchors (APs) in the network. The performance analysis in such an environment has been compared among various anchor (AP) deployment mechanisms; Figure 14 and Figure 15 illustrate the performance evaluation for a uniform, around the perimeter and random deployment mechanism and for both algorithms, (SF-MFMPL) and (C-MFMPL), respectively.

In accordance with the simulation results above, we have indicated the impact of the anchor node deployment, and it is clearly affected by the localization performance. A comparison of the performance evaluation for both proposed solutions, SF-MFMPL and C-MFMPL, under the two cases of the anchor node (AP) deployment mechanisms has been summarized in Table 5 and Table 6.

**Table 5:** Average localization error comparison of the SF-MFMPL proposed solution according to the anchor node deployment configuration.

Propagation Model Case	Average Localization Error (m)			
	SF-MFMPL Random Deployment		SF-MFMPL Perimeter Deployment	
	Lateration	Trilateration	Lateration	Trilateration
**Case I**	***7.86***	***6.76***	***4.34***	***3.86***
**Case II**	***8.25***	***6.91***	***4.67***	***4.19***

**Table 6:** Average localization error comparison of the C-MFMPL proposed solution according to the anchor node deployment configuration.

Propagation Model Case	Average Localization Error (m)			
	C-MFMPL Random Deployment		C-MFMPL Perimeter Deployment	
	Lateration	Trilateration	Lateration	Trilateration
**Case I**	***6.98***	***6.01***	***3.77***	***3.20***
**Case II**	***7.30***	***6.50***	***4.01***	***3.41***

According to the simulation results stated in Table 5 and Table 6, the localization performance of the uniform, around the perimeter deployment of the anchor nodes is better than the random deployment, as well as being the optimal placement configuration for an effective localization accuracy. In addition, the proposed algorithm C-MFMPL showed a better performance than the SF-MFMPL algorithm for both the lateration and the trilateration technique.

**Figure 16:**
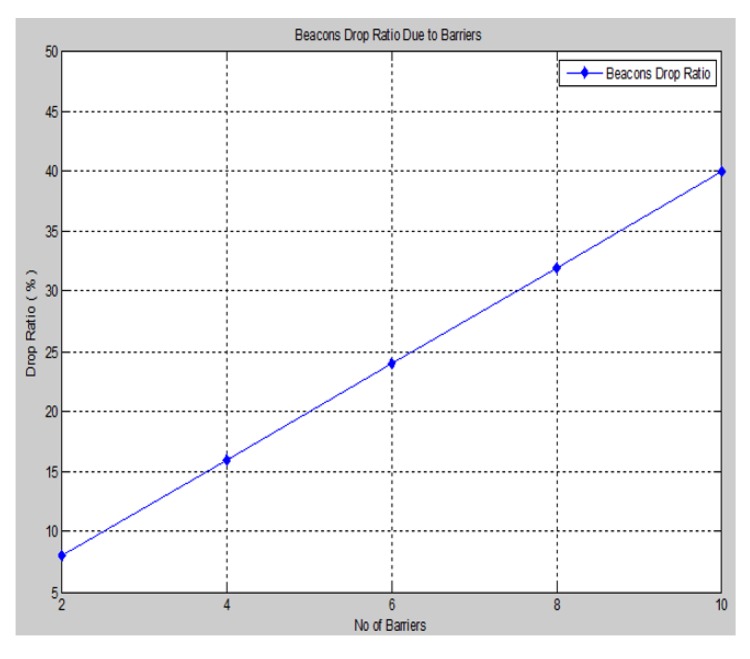
Invalid beacon drop ratio *vs.* No. of barriers.

Another important metric that indicates the effect of the barrier attack on the localization performance is the percentage of the invalid beacon drop ration due to the barrier attenuation of the signal with respect of the total percentage of available beacons for the proposed system, as shown in Figure 16.

We observe from the figure above that as the number of barriers increases, a greater number of invalid beacons will appear in the network, and drop ratio is increased, as well. In spite of the 40% invalid beacon drop rate from the case of 10 barriers, the performance of our proposed approach is still accurate and contributes to a lesser localization error.

**Figure 17:**
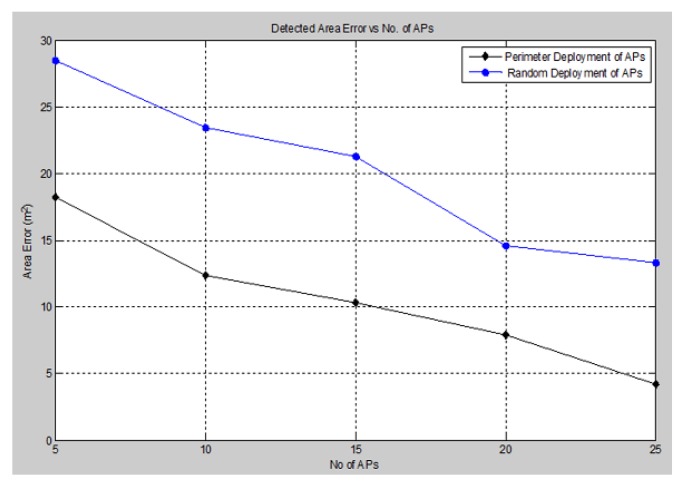
Detected area error *vs.* No. of APs.

One of the most significant performance evaluation criteria is the accuracy of the detected area of attack. This accuracy is measured in terms of the difference between the actual area occupied by the barrier and the detected area of the signal holes. We varied the number of anchors (APs), as well as the deployment configuration in order to determine the optimum placement positions for the detection mechanism. The accuracy of the detected area of attack is illustrated in Figure 17.

Based on the simulation results above, we observe that as the number of anchors (APs) increases, the detected area error reduces greatly, as well as the optimum placement configuration of anchors (APs) around the perimeter showed more accurate performance through identifying the attack area with great precision.

**Figure 18:**
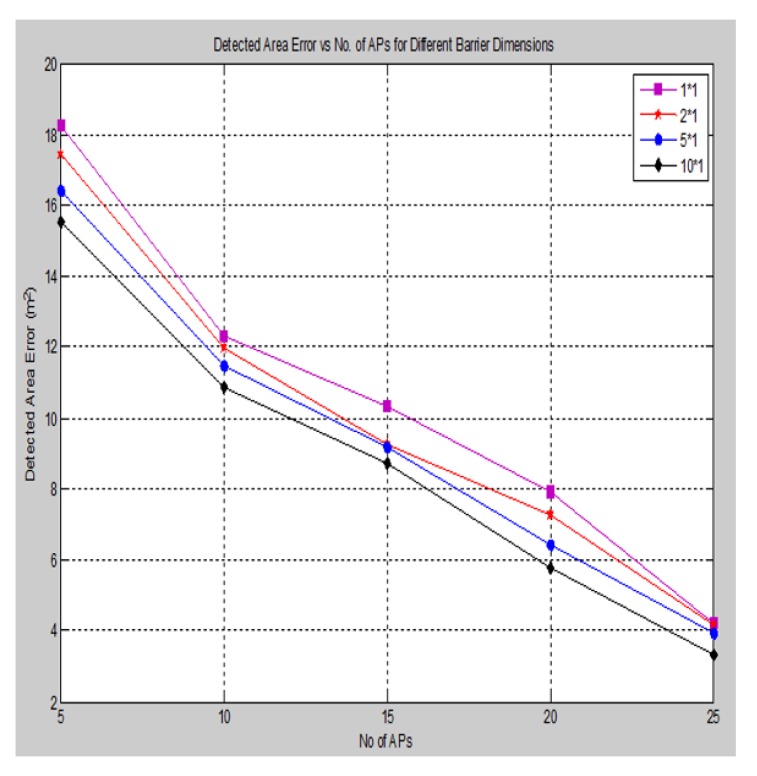
Detected area error for different barrier dimensions.

The accuracy of detecting the area of attack also depends on the shape and dimension of the barrier attack that is introduced into the network. We introduced a rectangular barrier of various dimensions and measured the accuracy of the detected area of attack, as shown in Figure 18.

In accordance with the simulation results above, initially, when the barrier size increases, the error in the detected area of attack is decreased gradually with respect to the increasing of the anchors (APs); this is due to more beacon signals being affected by these barriers with more RSS error deviations having been neglected, so that there is more information available for detecting the area of attack. On the other hand, increasing the barrier size will lead to increasing the localization error slightly.

## 7. Conclusions

This paper presents the robust range-based RSSI localization algorithm in the presence of barrier attacks and under the log-normal shadow fading model. These attacks affect the localization process, making it erroneous. The proposed algorithm has been achieved by two techniques of multiple frequencies and power levels identified by (SF-MFMPL) and (C-MFMPL), respectively, with averaging the received power levels all over the transmitted frequencies in order to mitigate the shadowing effects in the wireless channel propagation; besides, our solution includes lateration- and trilateration-based techniques and fingerprint matching, which were demonstrated to be efficient and accurate localization techniques for wireless sensor networks. Furthermore, in this paper, the detection and identification of the attack area have been achieved through applying a grid coloring algorithm with the aid of the approximate angle of arrival estimation of the received signal from each anchor (AP). Moreover, a suitable choice of the threshold error value between the actual and the estimated received signal strength was made. By identifying the attack area, the network operators take action to clear this attack and improve the localization accuracy. Moreover, the important key role of this paper is the trade-off between the localization enhancement and the utilization of a minimum number of anchor nodes with the best deployment. Through simulation results, we have proven the effectiveness of our approach. Future work directions for a real implementation and experiment design will be taken into consideration for testing the proposed algorithms.
